# Is Cholesteatoma a Risk Factor for Graft Success Rate in Chronic Otitis Media Surgery?

**Published:** 2015-11

**Authors:** Mohammad Faramarzi, Mohammad Mehdi Dehbozorgi, Seyed Taghi Heydari

**Affiliations:** 1 *Department of Otolaryngology, Head and Neck Surgery, School of Medicine, Shiraz University of Medical Sciences, Shiraz, Iran.*; 2 *Health Policy Research Center, School of Medicine, Shiraz University of Medical Sciences, Shiraz, Iran.*

**Keywords:** Chronic Otitis Media, Cholesteatoma, Graft Success Rate

## Abstract

**Introduction:**

In developing countries, chronic otitis media (COM) and cholesteatoma are relatively prevalent. Within the field of otology, COM surgery remains one of the most common surgical treatments. Most recent studies evaluating the potential prognostic factors in COM surgery have addressed graft success rate and types of middle ear and mastoid pathology. There has been much controversy about this issue until the present time. This study evaluated the effect of cholesteatoma on the GSR in COM surgery.

**Materials and Methods:**

The present retrospective, case-controlled study investigated 422 ears undergoing COM surgery. The minimum and maximum postoperative follow-up periods were 6 and 48 months, respectively. The study group consisted of patients with cholesteatomatous COM, while the control group included patients with non-cholesteatomatous COM, who had undergone ear surgery. Postoperative graft success rate and audiological test results were recorded and the effect of cholesteatoma on graft success rate was investigated.

**Results:**

The overall GSR was 92.4%. In the study group (COM with cholesteatoma),the postoperative GSR, mean speech reception threshold improvement, and mean air-bone gap gain were 95.3%, 2.1 dB, and 3.2 dB, respectively. In the control group (COM without cholesteatoma), however, these measurements were 90.9%, 9.4 dB, and 9.1 dB, respectively. The difference between the two groups was not statistically significant.

**Conclusion:**

The study results suggest that cholesteatoma is not a significant prognostic factor in graft success rate.

## Introduction

Chronic otitis media (COM) is one of the most common otological diseases in the developing world. COM is defined as persistent infection or inflammation of the middle ear and mastoid air cells. It typically involves a perforation of the tympanic membrane with intermittent or continuous otorrhea. This condition increases the likelihood of cholesteatoma formation (1). Histologically, cholesteatoma associated with COM is a destructive lesion that contains fibroblasts, keratinocytes, and inflammatory cells originating from squamous epithelium, which affects the middle ear, mastoid cavity, or both (2). The true incidence of cholesteatoma is unknown; nonetheless, retrospective data suggest a mean annual incidence of 9.2 per 100.000 individuals across all age groups (3). The most common presenting features of this disorder include otorrhea, hearing loss and, less frequently, dizziness. Possible complications include dizziness, deafness, facial nerve paralysis, labyrinthitis, meningitis, and brain abscess (4). Acquired cholesteatoma and other middle ear pathologies, such as granulation tissue, polyps, and hypertrophic mucosa can often develop as a result of COM.

Many reports in the literature discuss the prognostic factors affecting the tympanic membrane graft success rate (GSR) after tympanoplasty and tympanomastoid surgeries. One of the developments in this field is Becvarovski- Kartush's modification of the Middle Ear Risk Index (MERI) which includes seven variables; i.e., otorrhea, perforation, cholesteatoma, ossicular status, middle ear status, previous surgery and smoking, as factors affecting the prognosis post tympanomastoid surgery (5). 

Researchers have also evaluated other prognostic factors of GSR, including the site and size of tympanic membrane perforation, age, normal Eustachian tube function, graft material, mastoidectomy, surgical technique and the surgeon’s level of experience(6-12). However, other investigators have concluded that there were no significant predictors of successful graft outcome after tympanoplasty (13-15). Thus, the present study aims to evaluate cholesteatoma as a possible prognostic factor for GSR. 

## Materials and Methods

The present retrospective, case-controlled study aimed to investigate 422 ears that had undergone COM surgery. This research was approved by our institutional review board. All the surgical procedures were performed by an academic otolaryngologist in Dastgheib Hospital, a tertiary referral center affiliated to Shiraz University of Medical Sciences, Shiraz, from 2003 to 2012. All patients included in the study were adults aged between 15 and 65 years. All operations were performed by the first author. Success was defined as an intact tympanic membrane graft in the correct position together with a well-aerated mesotympanum. Postoperative follow-up assessments were carried out between 6 and 48 months. Patients whose ears were examined by microscopic otoscopy together with postoperative findings were recorded in questionnaires. Pure tone audiometry (PTA) and air-bone gap (ABG) were analyzed at 500 Hz to 4 KHz. Speech reception threshold (SRT), mean air conduction (AC), bone conduction (BC) and ABG gain were also analyzed. A patient was termed as having mixed hearing loss if they had a BC threshold exceeding 20 dB associated with an ABG of ≥ 15dB in at least two subsequent frequencies. In order to be included in the study, each patient had to have been followed up for at least 6 months post surgery. Study exclusion criteria were: suffering from ischemic heart disease (hypertension, diabetes mellitus or ischemic).

 In this study, a post-auricular approach with underlay tympanoplasty technique was used and the temporalis fascia was harvested for graft material. Types of operations including tympanoplasty, intact canal wall (ICW) tympanomastoidectomy and canal wall down (CWD) tympanomastoidectomy, were determined based on the preoperative and intraoperative findings. All the patients had a greater than 50% perforation of the tympanic membrane surface due to COM. In all CWD tympanomastoidectomy cases, obliteration of the mastoid cavity and reconstruction of the posterior canal wall was performed with the posteroinferior and or anterosuperior base muscle flaps. In addition to this, conchal cartilage grafts from the site of the meatoplasty were used posterior to muscle flaps if muscle bulk was adequate. A silastic sheet was inserted over the promontory in all cases with where ossicular necrosis had occurred. Patients who had a confirmed diagnosis of cholesteatoma were considered as the study group, while patients without cholesteatoma were assigned to the control group, taking into account the exclusion criteria into account.

*Statistical analysis*

Statistical analyses were performed using SPSS software version 15.0. Descriptive variables such as mean and standard deviation were used. A Chi square test was performed for comparisons of categorical variables and independent-sample and paired T-tests were performed for continuous variables between two groups of patients. The study group included patients with COM with cholesteatoma and the control group included patients with COM but without cholesteatoma. P-values less than 0.05 were considered significant.

## Results

Unilateral and bilateral ear involvement in the study group was detected in 129(87.2%) and 19(12.8%) patients, respectively. In the control group, unilateral and bilateral ear involvement was found in 214(78.1%) and 60(21.9%) of patients, respectively. The difference between the incidence of unilateral and bilateral ear involvement between the study and the control group was statistically significant (P=0.023).

In the study and control groups, 11 (7.4%) and 180(65.7%) patients underwent tympanoplasty, respectively. Eight (5.4%) and 47(17.2%) patients underwent the ICW procedure in the study and control groups, respectively. Also, 129(87.2%) and 47(17.2%) patients underwent CWD tympanomastoidectomy in the study and the control groups, respectively. The most common type of operation was CWD tympanomastoidectomy in the study group and tympanoplasty in the control group (P<0.001). 

Only 5.4% of patients in the study group had normal ossicular chain, while this ratio was 61.3% in the control group. The overall GSR was 92.4%(390). GSR was 95.3% (141) in the study group and 90.9% (249) in the control group; however, the difference was not statistically significant (P=0.104). 

There was no recurrent cholesteatoma in the follow-up period. It may be that a longer follow-up period is needed to identify cases with recurrent cholesteatoma.

*Hearing results*

Pre-operatively, 127 (85.8%) of control and 21(14.2%) of study group patients suffered from conductive and mixed hearing loss, respectively. In addition, conductive and mixed hearing losses were respectively detected in 243 (88.7%) and 31(11.3%) patients in the control group (P=0.391). Post-operatively, three (2.0%), 119 (80.4%), and 26(17.6%) patients in the control group had normal hearing, conductive hearing loss, and mixed hearing loss, respectively.

By contrast, normal hearing, conductive hearing loss, and mixed hearing loss were detected in 33 (12.0%), 207(75.5%), and 34(12.4%) control group patients, respectively (P=0.001). In the frequency range of 500–3000 Hz, and separately at 4000 Hz, the mean preoperative and postoperative AC and BC in the study and control groups were investigated and are shown in (Table.1).

**Table 1:** Pre-operative and post-operative mean AC & BC in the frequency range of 500-3000 Hz and at 4000Hz between case and control groups

**Groups**		**Frequencies of 500-3000Hz**		**Frequency of 4000Hz**	
		Pre op (mean)dB	Post op (mean)dB	Pre op (mean)dB	Post op (mean)dB
AC	Case	47.2±15.7	44.2±15.7	50.9±17.5	51.1±19.8
	Control	40.2±15.2	30.1±17.1	44.6±19.1	38.8±22.1
	P value	<0.001	<0.001	0.001	<0.001
BC	Case	10.8±11.0	11.4±10.2	14.3±14.1	16.2±14.1
	Control	10.0±8.5	9.4±9.2	14.5±13.9	16.1±15.5
	P value	0.387	0.042	0.880	0.937

AC=air conduction, BC= bone conduction

The mean preoperative and postoperative SRT and ABG were investigated and are shown in (Table.2). 

**Table 2:** Pre-operative and post-operative mean SRT, ABG and SRT and ABG difference in the case and control groups.

**Groups**	**Pre op mean SRT (dB )**	**Pre op mean ABG (dB )**	**Post op mean SRT (dB )**	**Post op mean ABG (dB )**	**SRT diff** **(dB )**	**ABG diff** **(dB )**
Case	47.0±15.8	36.4±12.2	44.9±16.9	33.2±11.0	2.1±15.5	3.2±12.2
Control	40.3±15.1	30.2±11.4	30.9±17.6	21.1±12.3	9.4±16.8	9.1±13.5
P value	<0.001	<0.001	<0.001	<0.001	<0.001	<0.001

SRT=speech reception threshold, ABG= air-bone gap

## Discussion

Surgery is the mainstay of treatment in COM with cholesteatoma (4). Removal of cholesteatoma, creating a dry ear and intact tympanic membrane, eradication of disease and prevention of complications are the key aims of surgery (16-18).

There is debate in the literature as to whether or not cholesteatoma has any effect on the GSR after COM surgery. Some studies have evaluated the effect of cholesteatoma on GSR. The findings of the present study suggest that cholesteatoma is not a risk factor for COM surgery. Some authors have shown that it may play an important role in graft take success. Moreover, Becvarovski and Kartush and Pinar et al. revised and updated MERI and considered middle ear condition and cholesteatoma as risk factors for GSR (5,19). Furthermore, Gersdorff et al. concluded that wet ears were associated with a high rate of re-perforation which influenced surgical outcome (20). In contrast, Baylan et al. stated that the condition of the middle ear at the time of surgery did not affect the GSR (21). Merchant et al. evaluated the efficacy of tympanomastoid surgery in active COM in two cholesteatoma and granulation study groups and achieved 89% GSR in 272 cases (18). Also, they concluded that COM with cholesteatoma had a significantly better outcome compared to COM with granulation tissue.

In a retrospective review of 73 patients, Yaor et al. concluded that surgery for COM was successful in both cholesteatoma and non-cholesteatomatous diseases (22), achieving 89% GSR.

Cruz et al. studied the efficacy of surgical treatment of COM with cholesteatoma in 36 patients (39 ears) and concluded that either ICW or CWD mastoidectomy with tympanoplasty gave satisfactory results regarding the cholesteatoma control (2). They did not highlight GSR in their series.

Ordonez et al. retrospectively evaluated the risk factors leading to failure in tympanoplasty in 280 years and concluded that cholesteatoma was not a risk factor (13). This study included 94(33.6%) primary cholesteatoma and 20(28.6%) revision cholesteatoma cases. The follow-up period and the number of surgeons were not specified.

In this study, the overall GSR was 92.4%, which is consistent with other studies reporting success rates of 60–99% (6). GSR was 95.3% in the cholesteatoma group and 90.9% in the non-cholesteatoma group. The difference between the two groups was not statistically significant.

Hearing results were also acceptable in our study. In the cholesteatoma group, there was a 2.1 dB SRT improvement and 3.2 dB gain for ABG closure. In the non-cholesteatoma group, there was a 9.4 dB SRT improvement and 9.1 dB gain for ABG closure. Cruz et al. (2) used SRT as a more reliable tool for functional hearing results and found that the ICW group maintained the same level of SRT and the CWD group had a slight increase (4 dB) in postoperative SRT. Moreover, Sarker et al. stated that hearing improvement was greater in dry perforation (6).

It should be emphasized that the limitation of current study is its nonrandomized, retrospective method. Since every surgical technique has its own precise indication, is considered to be ethically unfeasible. In addition, the other limitation is that no other factors (possibly confounding factors) have been evaluated. Various prognostic factors have been reported in the literature and there is a risk that the absence of any relationship shown in this study is in fact distorted because the prognosis also correlated with other factors. Systemic diseases, young and old patients, smokers, revision surgeries, and ears involved with tympanosclerotic plaques and granulation tissues were excluded from the study. Thus in order to evaluate the role of all factors, multicenter, prospective studies with a multiple logistic regression analysis are needed.

## Conclusion

The findings of the present study show that cholesteatoma is not an important prognostic factor in graft success rate.
